# 3D Structure From 2D Microscopy Images Using Deep Learning

**DOI:** 10.3389/fbinf.2021.740342

**Published:** 2021-10-28

**Authors:** Benjamin Blundell, Christian Sieben, Suliana Manley, Ed Rosten, QueeLim Ch’ng, Susan Cox

**Affiliations:** ^1^ Centre for Developmental Biology, Institute of Psychiatry, Psychology and Neuroscience, King’s College London, London, United Kingdom; ^2^ Nanoscale Infection Biology Lab (NIBI), Helmholtz Centre for Infection Research, London, Germany; ^3^ École Polytechnique Fédérale de Lausanne, Lausanne, Switzerland; ^4^ Snap, Inc., London, United Kingdom; ^5^ Randall Centre for Cell and Molecular Biophysics, King’s College London, London, United Kingdom

**Keywords:** SMLM, deep-learning, structure, storm, AI

## Abstract

Understanding the structure of a protein complex is crucial in determining its function. However, retrieving accurate 3D structures from microscopy images is highly challenging, particularly as many imaging modalities are two-dimensional. Recent advances in Artificial Intelligence have been applied to this problem, primarily using voxel based approaches to analyse sets of electron microscopy images. Here we present a deep learning solution for reconstructing the protein complexes from a number of 2D single molecule localization microscopy images, with the solution being completely unconstrained. Our convolutional neural network coupled with a differentiable renderer predicts pose and derives a single structure. After training, the network is discarded, with the output of this method being a structural model which fits the data-set. We demonstrate the performance of our system on two protein complexes: CEP152 (which comprises part of the proximal toroid of the centriole) and centrioles.

## 1 Introduction

Imaging mesoscale 3D biological structures (that is, those between the nano- and the micro-scale) is a critical problem in biology, as many processes of biological interest rely on collections of proteins or other molecules arranged into a distinct architecture. Currently two major techniques can provide data on the shape of such aggregates: electron microscopy and light (particularly fluorescence) microscopy. Electron microscopy (EM) offers resolution below 1 nm, but is limited in the thickness of the samples it can observe, and analysis is relatively complex, generally requiring multiple particle averaging (Milne et al., 2013). Fluorescence microscopy is experimentally relatively simple and can deal with larger samples, but generally yields only single images which are limited in resolution to about 250 nm (Schermelleh et al., 2010).

Super-resolution techniques allow this limit to be broken, pushing the achievable resolution down to 20–100 nm. In particular, single molecule localisation microscopy (SMLM) yields high resolution images (around 20–30 nm), while allowing large amounts of data to be collected (Schermelleh et al., 2010; Holden et al., 2014) and being relatively experimentally simple. SMLM imaging has a trade off between the *x*, *y* and *z* resolution: gaining information in the *z* direction is possible, but generally at the expense of in-plane information quality (Badieirostami et al., 2010). Therefore, 2D images will have the highest localisation quality, but clearly limit information on 3D structure.

The challenge of how to infer 3D information from 2D images has been tackled both from the perspective of synthesising EM images to create a 3D structural model (Milne et al., 2013), and in the computer vision field to infer a 3D structure from a single image of a single object (Fan et al., 2017). In recent years, deep learning has emerged as a promising approach to improve structural fitting.

Convolutional neural networks are one of the most well known forms of Deep Learning - convolving the data with a kernel (Goodfellow et al., 2016). This process reduces the size of the principal data dimensions, creating a number of feature maps or filters, each sensitive to a particular, local aspect of the data. Through training, the network parameters adjust to produce the required output.

Here, we use a deep learning network to infer the pose of point cloud data and 3D structure. Our algorithm HOLLy (Hypothesised Object from Light Localisations) allows us to perform a completely unconstrained model fit from 2D SMLM images.

## 2 Methods

### 2.1 Modelling Pose Using Deep Learning

HOLLy fits a 3D model against a set of 2D images of the same biological structure. The input images are typically super-resolved SMLM reconstructions, each of which is a z projection of the structure being imaged from some unknown rotational orientation and translation. The goal is to deduce the pose (rotation and translation) for each input image and infer a single 3D model for the entire data-set.

The 3D model is a collection of points (with their co-ordinates represented by a matrix) which are initiated at random positions. The current positions of the points, and the pose corresponding to each input image, are used to generate a simulated microscopy image corresponding to each input image (with the image being projected in z into a single x-y plane). The image is rendered with a Gaussian at each point, as is standard for SMLM. Each Gaussian has the same sigma, which is a parameter of the renderer, and the resulting image is differentiable with respect to the point coordinates and sigma. Our renderer is designed to efficiently and accurately render SMLM point clouds. This is in contrast to existing state of the art such as OpenDR (Loper and Black, 2014), DiRT (Henderson and Ferrari, 2020), PyTorch3D (Ravi et al., 2020), Pulsar (Lassner and Zollhöfer, 2020) and DWDR (Han et al., 2020) which are designed primarily to render illuminated, textured meshes with perspective cameras (or in the case of Pulsar and Insafutdinov and Dosovitskiy (2018), rendering with spheres), our renderer is simpler and more closely models SMLM. Rather than rendering rasterised triangles, HOLLy converts the final 2D points to Gaussians.

**FIGURE 1:**
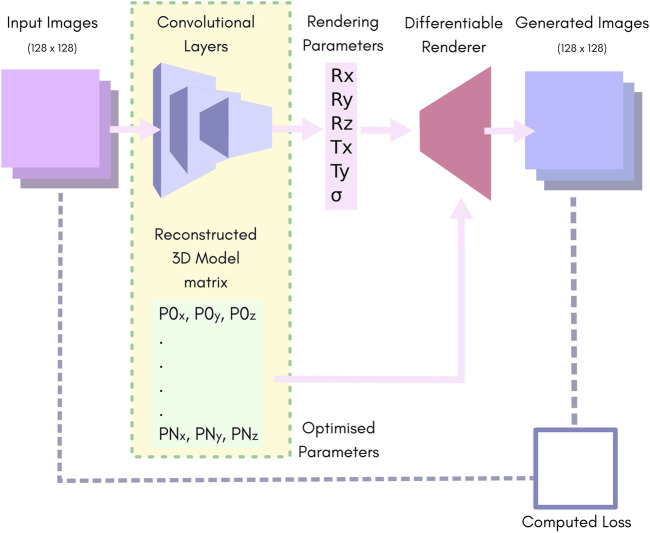
An overview of our network. Batches of images of size 128 × 128 pixels are fed to the convolution layers, which reduce the batch down to 6 parameters. These are passed to the rendering pipeline along with the 3D reconstruction matrix to produce a batch of output images. The rendering parameters Rx, Ry and Rz represent the rotation in “Angle-Axis” form. Tx and Ty represent translation in the X, Y plane. S represents the predicted output-sigma. The 3D reconstruction matrix contains a list of vertices representing the predicted point-cloud.

We used a simple convolutional neural network (CNN) consisting of 10 layers of strided convolutions and Leaky-ReLU (Aggarwal, 2018), followed by two fully connected layers. Figure 1 highlights the major components (further information can be found in Supplementary Material: HOLLy technical details).

The CNN yields six outputs. The position and orientation of the model are described by the translation in *X* and *Y*, and three rotation parameters for which we used the axis-angle formulation. The sixth parameter is the output-sigma value which is used as the sigma for the renderer. Note that the output-sigma explicitly differs from the resolution of the input images, i.e., in the case where input images are themselves reconstructions of SMLM data, the sigma used for their reconstruction (*input-sigma*) is not the same as output-sigma.

In principle, if the input data were perfect, the output sigma could be fixed to be equal to the input sigma. Since this is not the case, allowing the model to predict output-sigma allows it to account for some of the noise in the data. For example, consider the case of scatter (noise in the position of fluorophores). That is essentially a stochastic blur of the model structure, so when the reconstructed 3D model (which has no scatter) is rendered, the sigma needs to be higher in order for the output to be a good match to the input. This is discussed further in Section 2.6.

The key element of our system is the use of a CNN to predict the pose for each input image. Allowing for a pose per image is a significant advantage over techniques such as template matching based Cryo-EM (Milne et al., 2013), or classification of the images by view (Salas et al., 2017) since the system is not limited to a small number of orientations, and views do not have to be determined a-priori on unknown structure in order to build a classifier.

**FIGURE 2:**
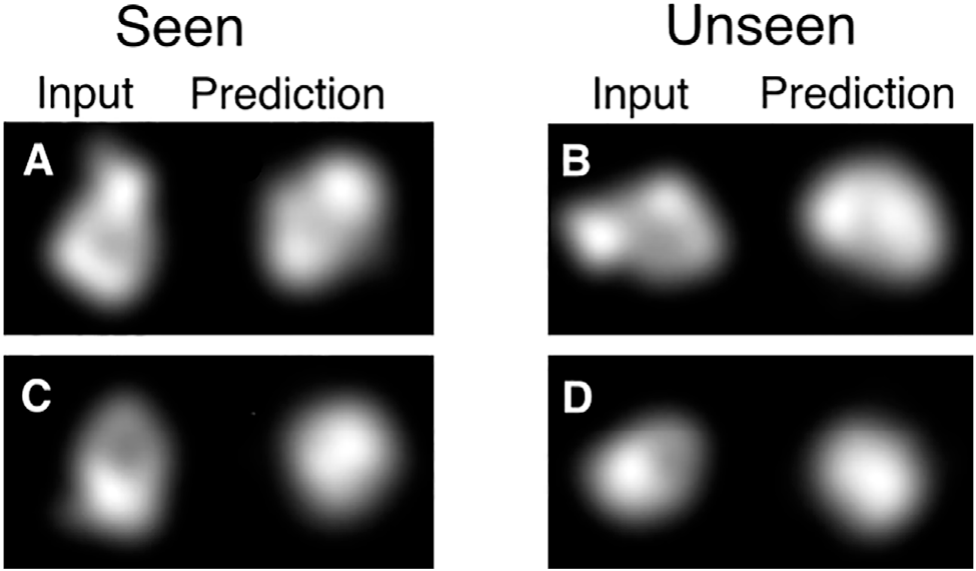
Demonstration of information sharing between different poses with the CNN. HOLLy was stopped half-way through the first epoch of training, meaning only half of the data has been used for training. Note that for the half of the data which has been seen, this corresponds to a single step of gradient descent per image, and half of the data has not yet been used at all. Already it can be observed that in many cases on seen data (illustrated in **A, C**) there is correspondence between the input and output shapes (albeit imperfect as it is very early in the optimization process). The advantage of the CNN can be observed in the results on unseen data where this correspondence also exists **(B, D)**. This partial convergence on unseen data shows that the CNN allows earlier data to assist in the convergence of data seen later, which provides a very substantial improvement over modelling poses separately.

Additionally, using a CNN to predict the pose has a big advantage in modelling a pose per image as it makes the overall optimization much more tractable. The reason for this is that the space is in some sense smooth and images that are close in appearance will usually also be close in pose. This allows the network to aggregate information from similar images in order to get a better prediction of the pose for all of them. It also allows for fast convergence because an improvement on one image can cause an improvement in many others. We illustrate this in Figure 2, where data that is not seen during training can generate outputs that correspond to the input.

The advantage of using a CNN can be illustrated by attempting to solve the same problem by direct optimisation. We removed the convolutional layers from the architecture shown in Figure 1, replacing them with a single 5 × *N* matrix (*N* being the size of the training set). A training batch consists of a batch of images and their corresponding poses from that 5 × *N* matrix. These differentiable render is used to render the model with these poses. This rendered images are compared to the corresponding input image creating a loss as before. The loss is back-propagated through the diffentiable renderer and used to update the model and the poses. Various learning rates, models and optimisers were tested.

This direct optimisation approach could not reproduce 3D sample structure or model the pose correctly. We suspect this is due to both a lack of shared rotational model between data and the difficulty of modelling rotation. The results can be found in Supplementary Material: Direct Optimisation. These results demonstrate the advantages of using a CNN in this scenario.

### 2.2 The Output Is a Structural Model Rather Than a Trained Network

Often, the value of a neural network is the network itself that can be used to predict, discriminate or otherwise solve a particular problem once trained. Our approach ignores the network once it has been trained; the value in our approach is the 3D model stored in the Reconstructed 3D Model matrix.

This 3D model gradually improves as training continues. The user can stop training at any time, typically when the loss stops improving. The final positions of the points in the 3D reconstruction matrix represent the final structure, whereupon the network is no longer required.

### 2.3 Simulated Data Models

In order to evaluate HOLLy, we selected a number of ground-truth point-clouds with different characteristics: a reduced version of the Stanford Bunny^1^, the Utah Teapot^2^ and an approximation of the CEP152/HsSAS-6 complex (Sieben et al., 2018).

All of the models consist of a relatively small number of vertices (fewer than 400). Each have unique characteristics, such as different numbers of vertices, symmetries and voids (see Section 3 - Results). The Stanford Bunny and Utah Teapot are standard in computer vision tests as they have properties that are likely to prove challenging. The Utah teapot is close to, but not quite, symmetric, and the Stanford bunny has fine structure (ears) but also relatively large areas of smooth structure (back). These properties showcase the potential of the method to yield results as experimental data improves.

As we have complete control of data-set generation from synthetic models, we must choose the distribution of data across the translation and rotation space. We uniformly sampled the 3D rotation group—SO(3)—which consists of all rotations in Euclidean $R^{3}$ space, centred at the origin, using the equation presented by^3^ Kirk (1994). The models used in the simulated data are small enough to be rendered “on-the-fly” into images as the network trains.

### 2.4 Experimental Data From Biological Structures

Our main biological targets were a centriolar complex comprised of the CEP152 protein, and purified centrioles.

The first dataset was a super-resolution (STORM) microscopy data-set of CEP152, obtained and analysed as described in Sieben et al. (2018). The structure of this centro-symmetric complex has been fitted with a toroid and found to be 400 nm in diameter (Sieben et al., 2018), which subsequent work confirmed (Kim et al., 2019). This yielded a list of localisations for each identified CEP152 structure which were reconstructed localisations into 2D images, rendering with a Gaussian. Since the number of localisations in the experimental SMLM data-sets outnumber the modelled point-cloud by a factor of 20, this process is computationally intensive, and so these images are pre-rendered and stored on disk.

This data-set consists of 4,663 individual images. Some of these show incomplete labelling or are not centriole structures (such as all the fluorophores converging on a single, bright spot). Erroneous data were removed manually, reducing the data-set size to 2055. Data was augmented by a factor of 20 rotating the entire centriole within the field of view using a 2D rotational matrix, giving a final training set size of around 40,000. As the data is represented by points and not a bitmap, it can be rotated by an arbitrary angle without introducing additional artefacts. Examples of the STORM CEP152 training images can be found in the Supplementary Figure S2.

The second data-set is derived from expansion microscopy experiments to image labelled glutamylated tubulin in centrioles purified from *Chlamydomoanas reinhardtii* (Mahecic et al., 2020). The images are segmented and presented as tiff stacks of size 128 × 128 × 84 in xyz. A sum projection is carried out to eliminate the information in z, creating a 2D image of an unknown blur. Each image was cropped to 60 × 60 pixels centred on the protein complex.

As the data are represented by pixels and not a list of localisations, augmentation is limited to the four cardinal directions to avoid the creation of artefacts. The resulting data-set is 14,612 items in size. As the point-spread function is not modelled, there is no base input-sigma. A Gaussian blur of decreasing sigma is applied “on-top-of” the existing image (see Supplementary Figure S3).

### 2.5 Input Images

The input to the network consists of a batch of 2D images, each of the same target object from different viewpoints. These images may be simulated (rendered from a known ground-truth 3D model) or derived from experimental data.

For both simulated and real SMLM data, rendering with a Gaussian generates a 2D image, with the resolution of the reconstruction being determined by the input-sigma. For the simulated data the 2D point cloud is generated by applying a random rotation and translation, adding noise and projecting away Z. For data in the form of images were blurred with a Gaussian, with input-sigma as the width.

Before being passed into the network, the input images were normalised to ensure that the pixel values fall within boundaries usable by the network (see Section 2.8).

Deep learning requires a large, representative training set for results to be accurate. For accurate 3D reconstruction, it is important to sample diverse angles since areas of the object not represented in the training data will not be reconstructed. In the simulated case, data-sets of any size can be generated (time permitting). However, this is not the case for the experimental data.

### 2.6 Sigma

The input-sigma value, which defines the level of blur (i.e., resolution) in the input images, is initialised at a high value (one which would produce an image with around diffraction limited resolution). The value is then decreased on a curve as the network trains. By starting with a larger input-sigma, the loss between the input and output images is smaller, with shallower gradients over larger distances. This allows the network to broadly optimise the points in the 3D reconstructed model matrix, refining finer detail as the input-sigma is reduced.

The lowest value for sigma can be set to the expected localization error for a particular SMLM experiment. The input-sigma curve can be found in the Supplementary Figure S1. The output-sigma (that is, the sigma used by the differentiable renderer to create images from the hypothesised model) is predicted by the network. The output-sigma can be set to match the known input-sigma, but early experiments suggest that predicting the output-sigma increases the network’s tolerance to scattered or missing fluorophores. By increasing the output-sigma the blur increases, accommodating the scattered points.

In experimental data, we would expect around an ∼8 nm scatter in position due to the antibody used and an additional ∼12 nm degradation in precision due to the localisation accuracy. Such values suggest an expected resolution around 20nm, with an expected sigma around 10 nm. For our STORM CEP152 experiments we set the lower-bound of the input-sigma to a value of ∼3.2 pixels, which equates to 30 nm using the scale provided with the data. The input-sigma changes at the end of each epoch, rather than continuously, giving a “stepped-curve” (see Supplementary Material: Note 3 - input-sigma Hyper-parameter and Supplementary Figure S1) This is due to the images being pre-rendered before training begins. This decision was made for performance reasons.

The expansion microscopy centriole data-set has a scale of 14 nm per pixel. The additional input sigma curve begins at 2.8 pixels (∼ 40 nm), reducing to zero. The smaller initial input sigma attempts to account for the smaller image size and the unknown resolution of the data.

### 2.7 Loss Function

The loss is calculated directly between images by comparing the pixel values between the input data and the predicted result, using the PyTorch L1 Reduction option^4^ with the ‘sum’ reduction. Rather than use the L1 loss between entire images, a mask was generated from target image. The loss was calculated only for these pixels that are within the mask, with areas outside the mask set to zero for both input and output images.

### 2.8 Reconstructed 3D Model Matrix and Normalisation

As training progresses, the matrix of 3D points that represents the reconstructed 3D model moves from a random positions to yield a recognisable structure. The size of this matrix (the number of points to optimise) is ultimately limited by the amount of memory and time available to the end user. The matrix size is chosen by the user before training starts. In simulated tests, the number of points responsible for generating the input image is generally known, except when multiple fluorophore reappearances per point are simulated.

**FIGURE 3:**
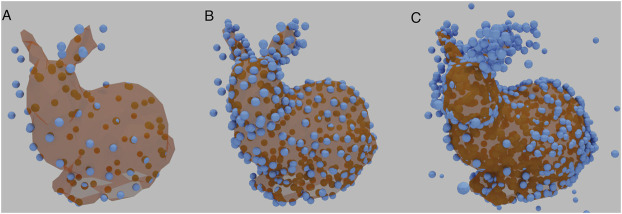
Examples of a reconstructed Stanford Bunny using different sizes of 3D reconstruction matrices. **(A)** contains 100 vertices. **(B)** contains 350 vertices, the same number as the underlying ground truth. **(C)** contains 1,000 vertices. HOLLy manages to reproduce the basic shape throughout.

Since the number of points affects the integrated intensity of the image, and thus the loss, the number of points is linked to the appropriate learning rate. Normalisation was therefore required to bring the training data into numerical ranges the network can process without generating extreme gradients. The image tensor was divided by the integrated intensity, followed with a multiplication by a fixed scalar. Figure 3 shows three examples of Stanford Bunny Reconstructions, each with a different size of 3D reconstruction matrix, with normalisation applied. In each case, the basic shape is recognisable, with increasing detail.

### 2.9 Hyper-Parameter Choices

Hyper-parameters are the user-chosen settings (Goodfellow et al., 2016), rather than the learned parameters. Our parameters were chosen using a combination of existing defaults and explorations within reasonable ranges.

To verify that the learning rate selected was appropriate the suggested value of 0.004 for the Adam Optimizer (Kingma and Ba, 2017) was varied by a factor of 10 in both directions, stopping when structure reproduction began to fail, with a score of 0.0004.

The simulated data-sets used comprised 40,000 images, generated from an initial set of 2000 images. Each image was augmented 20 times by a random rotation around the *Z* axis to better match the experimental data.

The number of images presented to the network at each training step (the batch-size) can affect the final accuracy of the network (Kandel and Castelli, 2020). A batch-size of 32 was selected as appropriate. Decreasing the batch size too far caused reproduction to suffer and increasing too far caused memory usage to become computationally limiting.

The final parameter considered was the number of epochs (that is, the training time). An epoch is completed when the network has processed the entire training set once. A range of number of epochs were tested, with a value of 40 being found to be an acceptable trade-off between accuracy and time.

This baseline for training with simulated data was chosen after a number of results from earlier tests, with the restrictions of the final experimental data in mind. The most important of these is the training set size and construction. Experiments with increasing the size of the simulated training set gave improved results, but we are restricted in the size of the real, experimental data. Therefore we chose to match the size of the experimental data-set when performing the simulated experiments.

Further details of these hyper-parameters used in our experiments are listed in Supplementary Material: Note 5.

### 2.10 Implementation

Experiments were carried out with a nVidia GeForce 2080Ti GPU. Training duration was around 8 h with the settings given as the baseline. Larger numbers of points in the reconstructed 3D model dramatically increased memory usage.

The estimated energy use to train a model is 2.1 kWh based on a measurement of 623.4 kWh over 166 days. In this period, 298 models were trained and evaluated. This was confirmed by cross-checking against the wattage of the GPU and the time spent to generate a model.

Further technical details may be found in Supplementary Material: HOLLy Technical Details.

## 3 Results

### 3.1 Evaluation Criteria

The 3D structure which the network attempts to reconstruct is represented as a point cloud with the coordinates of each point stored in the 3D reconstruction matrix. The network attempts to learn the orientation over time, and simultaneously improves its own internal representation of the 3D structure by comparing 2D renders of the point cloud against the training images.

The effectiveness of our approach was assessed by measuring the similarity between the input point-cloud and the resulting point-cloud stored in the model’s 3D reconstruction matrix. Finding the absolute best match between two structures is an NP-hard problem, and therefore a definitive score is not possible. Given this, we selected the root mean squared distance (RMSD) between two equivalent vertices in each point-cloud as an acceptable measure. Equivalence is determined by finding the closest neighbour with the Iterative Closest Point (ICP) (Arun et al., 1987) algorithm within CloudCompare^5^.

ICP relies on an rough, initial alignment. We performed this step manually, then applied ICP to obtain our RMSD score, independent of the pose predicted by the network. To find an RMSD score baseline to compare against we attempted to match two random clouds covering the same world-space as our model.

The parameters used in these experiments can be found in Supplementary Material: Experiment parameters.

### 3.2 Simulated Results

To assess the accuracy of our proposed method, a set of commonly used 3D models were chosen to evaluate the approach. The availability of a ground-truth structure allowed us to measure how well our network performs under different conditions. To validate our approach, we first performed a set of baseline experiments to determine how well the network could infer the 3D structure when only presented with 2D renders of these models.

#### 3.2.1 Baseline Experiments - Stanford Bunny

The first model tested was the Stanford Bunny. This model has no symmetry, contains fine detail, protrusions and a homogeneous distribution of vertices across its surface. It contains considerably more points than the other point-clouds used, though the version in our experiments is in the order of hundreds of vertices as opposed to tens of thousands in the original point-cloud.

**FIGURE 4:**
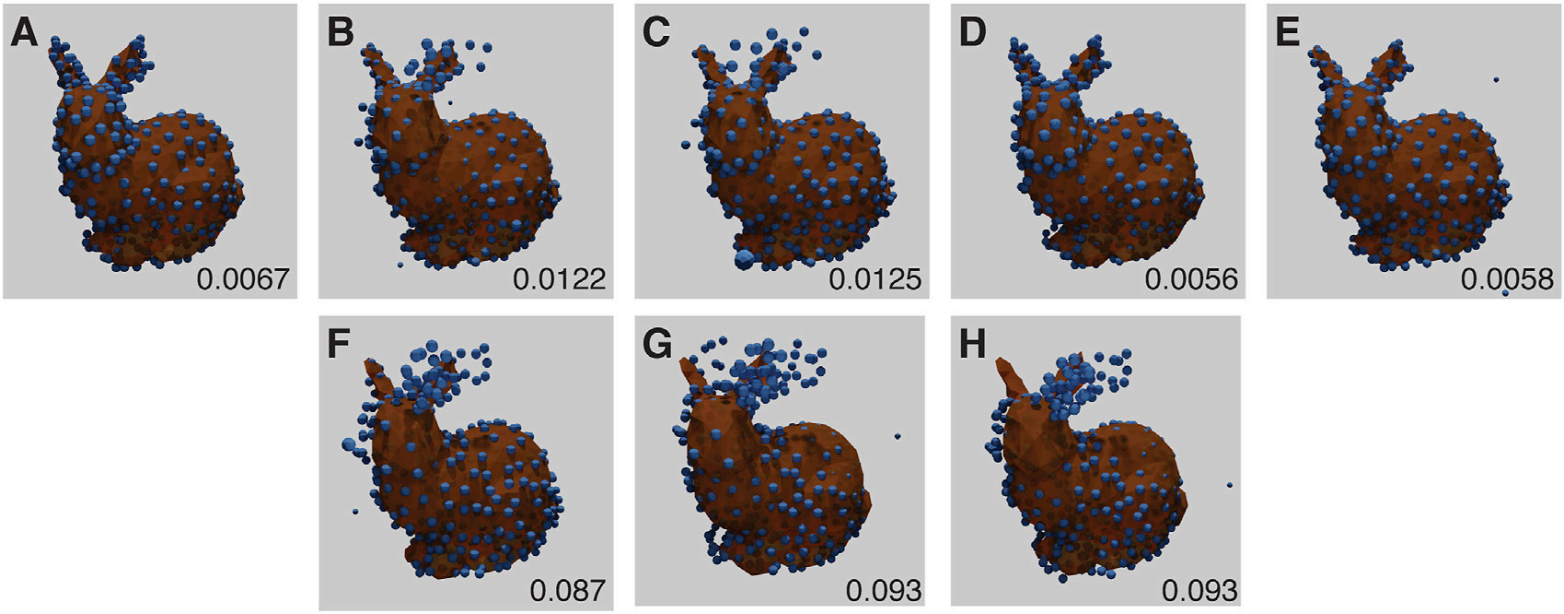
Baseline Stanford Bunny experiment results for each run **(A–E)** with RMSD score in the lower right corner of each panel. The ground truth model is shown in orange, with the inferred structure shown as blue spheres, overlaid and aligned. **(B–D)** have the models mirrored for display and RMSD computation, and show high quality fitting. **(F–H)** correspond to results for **(B–D)** as the original reconstructions (without mirroring). Note that under this imaging modality, the presence or absence of mirroring cannot be determined. The parameters for this experiment can be found in Supplementary Table S5.1.

All results from baseline experiments were noise free (i.e., every generated fluorophore was exactly at an existing vertex position, there was only one per vertex position, and every vertex position was occupied). The baseline results all had low RMSD scores, considerably less than 0.17, the average score when aligning two random point clouds of the same size (Figure 4). However, three of the runs showed a mirroring error, where the network mirrors the point-cloud in the dorsal plane. This is due to the lack of depth information in the training images (Figure 4), and is a fundamental ambiguity.

#### 3.2.2 Baseline Experiments - Utah Teapot

Our second choice of model was the Utah Teapot, which posed several challenges for our method: the similarity of the handle and spout (when rendered using points), the bilateral symmetry and the large voids between the layers of points in the central body.

**FIGURE 5:**
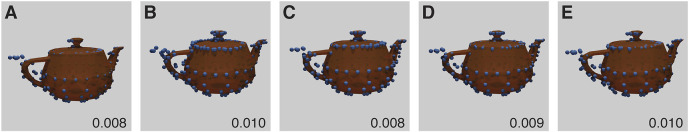
Baseline Utah Teapot Experiment results for each run **(A–E)** with RMSD score. The inferred structure shown as blue spheres, overlaid and aligned against the ground truth model shown in orange. Each model shows incorrect symmetry with non-differentiated spout and handle. The parameters for this experiment can be found in Supplementary Table S5.2.

It was reconstructed well and the pose was well predicted. However, the handle appeared to be the same as the spout. Both of these areas are low in information with few ground truth points. The predicted structure therefore has an additional transverse plane of symmetry not present in the ground-truth (Figure 5). From the tip of the spout, to the edge of the handle, the distance is 1036nm, using the CEP152 experiment scale.

#### 3.2.3 Baseline Experiments - Approximation of the CEP152/HsSAS-6 Complex

The third point-cloud used in these experiments is an approximation of the CEP152/HsSAS-6 complex (Sieben et al., 2018). The approximation consisted of two cylinders, one smaller and perpendicular to the other. This point-cloud is somewhat smaller than the others and is extremely regular with large gaps between the columns of points.

**FIGURE 6:**
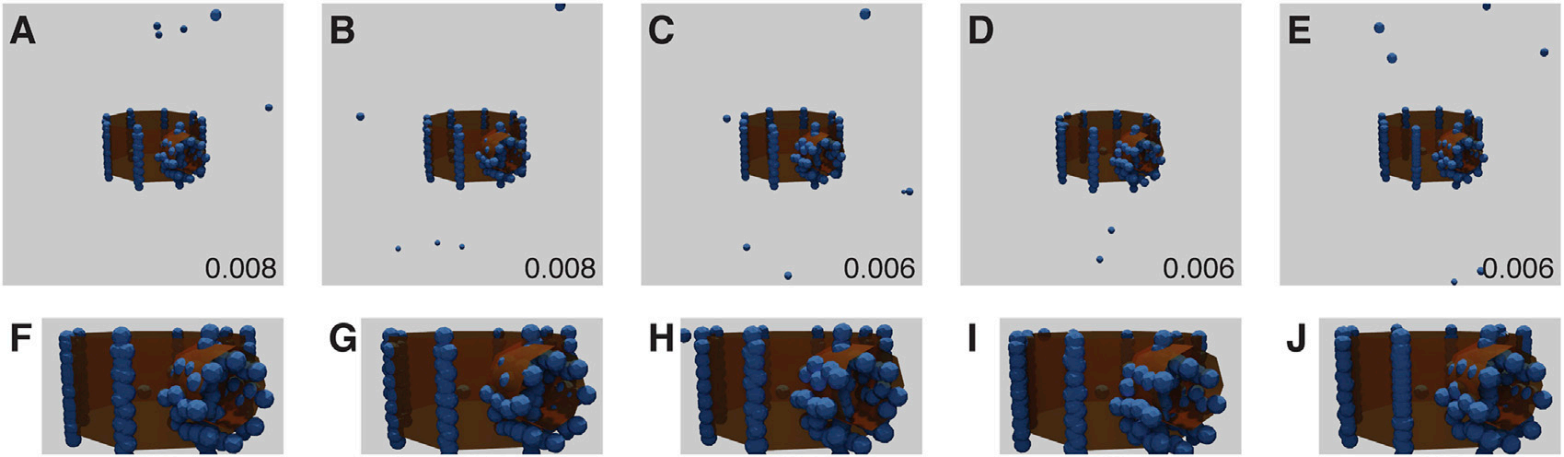
Baseline CEP152/HsSAS-6 approximation experiment results for each run (**(A–E)**, **top row**) with RMSD score. The ground truth model is shown in orange, with the inferred structure shown as blue spheres, overlaid and aligned. Bottom row **(F–J)**: close up of the **top** row. Note the slight offset of the smaller, reconstructed cylinder from the ground-truth. The parameters for this experiment can be found in Supplementary Table S5.3.

The smaller, cylindrical structure is offset towards the top of the larger structure in the ground-truth; this is not reflected in the reconstruction. This is likely due to the size of the point-cloud in the view - fine detail is hard to discern when the point-cloud is small (Figure 6). From the end of the small cylinder to the furthest edge of the larger cylinder, the distance is roughly 415 nm.

Together, these baseline experiments indicate that our approach is suitable for reconstructing the overall 3D structure from a series of 2D images. Most results showed low RMSD scores and produced structures that are a good match to the original 3D models.

### 3.3 Modelling Experimental Noise in Simulated Results

Our method aims to discern structure from fluorescence microscopy images, particularly super-resolution. We therefore focused on the kinds of problems often encountered in such experiments. Fluorophores are offset from the object they are labelling, they may not bind to certain areas, or might bind multiple times. They may not illuminate consistently or they may not be separable from their neighbours. We modelled three forms of experimental noise: missing fluorophores (where no fluorophores appear for a particular ground-truth point), scatter (where a fluorophore appears at a varying distance from its ground-truth point), and multiple binding (where multiple fluorophores appear for a single ground-truth point).

### 3.3.1 Scatter

Two factors can lead to scatter in fluorophore positions: the inaccuracy of the fitted position due to the limited number of photons collected, and the offset between the protein of interest and the label, with the largest effect arising from primary/secondary antibody labelling. This noisy change of position (scatter) is modelled using a random Gaussian distribution with a particular scatter-sigma value. The scatter-sigma ranges from 0.03 to 0.15 pixels (9–44 nm, given the scale in the CEP152 experimental data).

**FIGURE 7:**
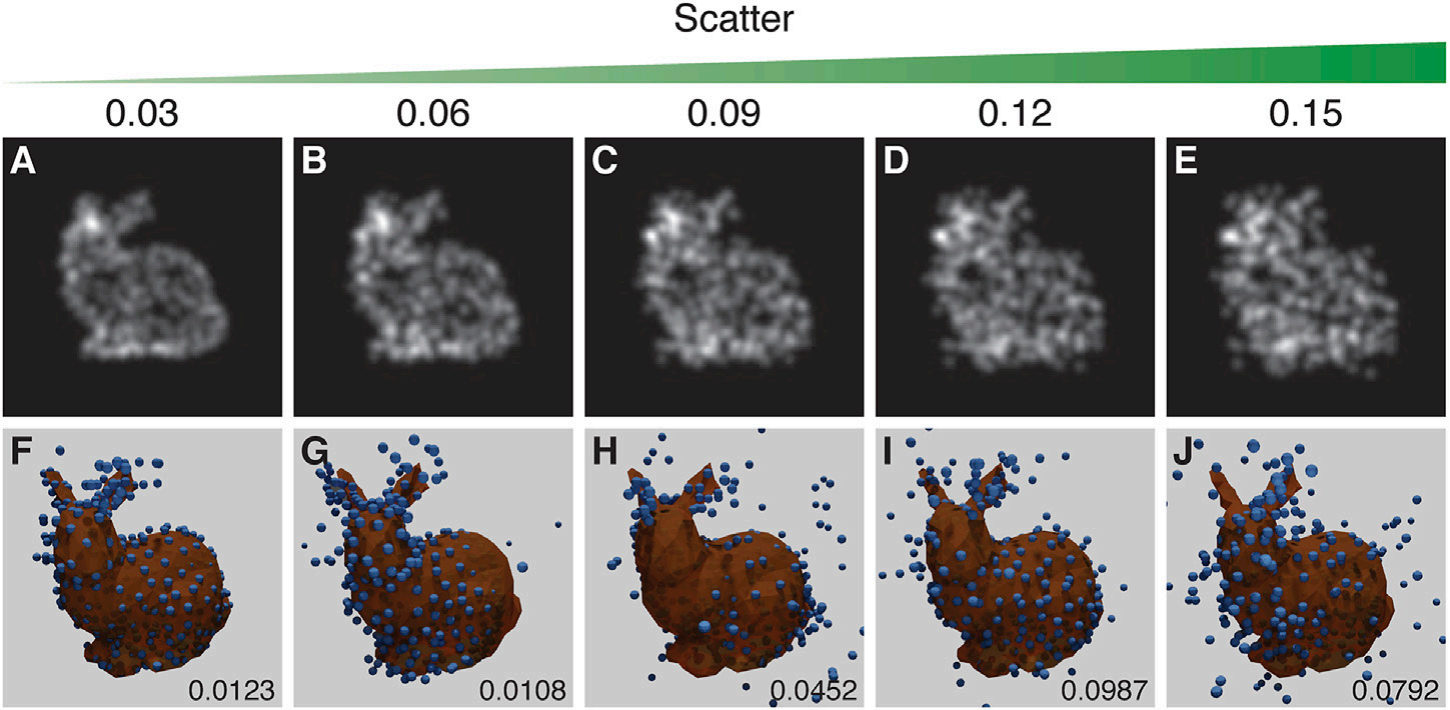
The results of the experiment into the effect of scatter. Top row **(A–E)**: examples of training images treated with increasing scatter, as indicated by the scatter value above each panel. Bottom row **(F–J)**: the corresponding results with the inferred structure shown as blue spheres, overlaid and aligned with the ground truth model shown in orange. RSMD scores are indicated for each run in the lower right corner of **(F–J)**. Runs in **(F)** and **(C)** respectively showed an incorrect symmetry in structure and mirroring in a vertical plane. The parameters for this experiment can be found in Supplementary Table S5.4.

The results suggest that a scatter-sigma value between 0.06 pixels and 0.09 pixels (20–29 nm) is the cut-off point for acceptable reproduction of the structure. The run in Figure 7F shows a rare error where the structure is symmetrical along the dorsal plane - effectively giving the structure two heads. Figure 7C suffers from the mirroring problem (Figure 7).

### 3.3.2 Missing Fluorophores

**FIGURE 8:**
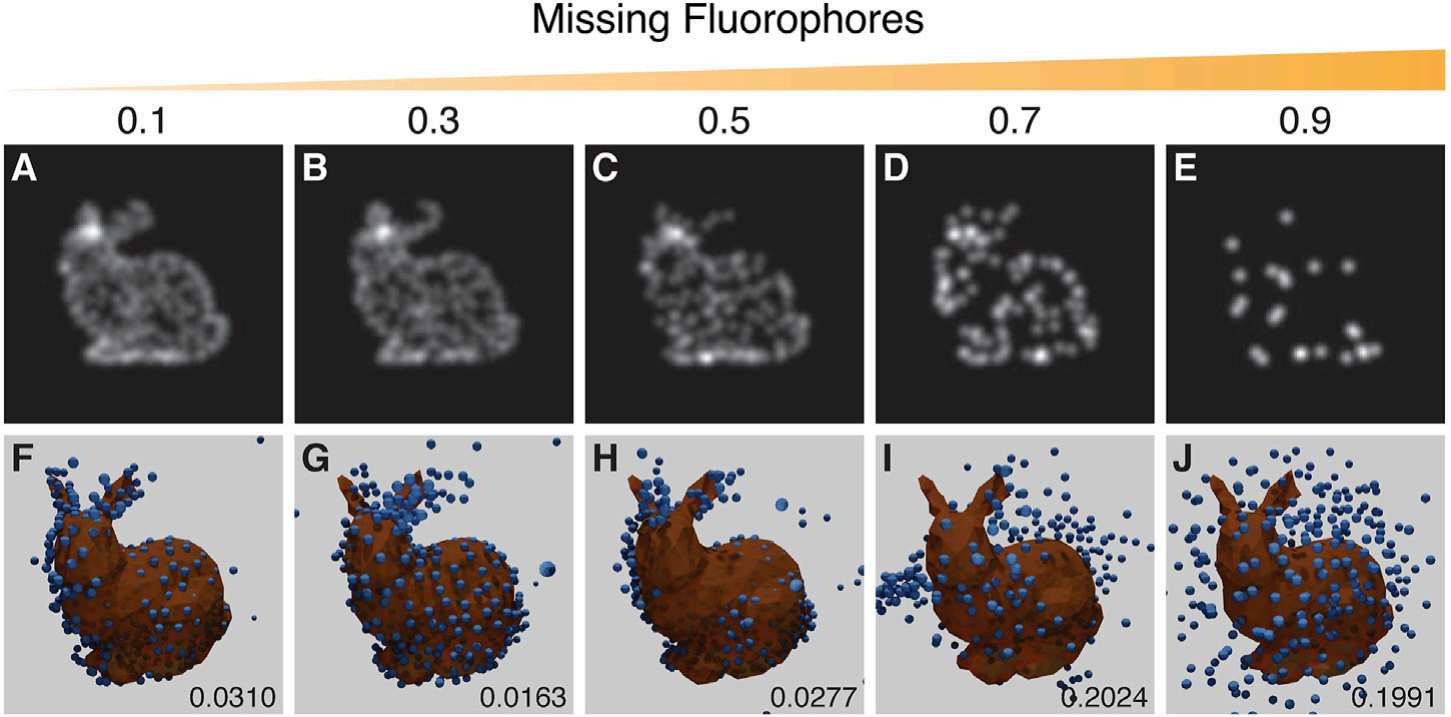
Results from the experiment on the effect of missing fluorophores. Top row **(A–E)**: examples of training images with increasing probability of removing points as indicated by the value above each panel. Bottom row **(F–J)**: the corresponding results with the inferred structure shown as blue spheres, overlaid and aligned with the ground truth model in orange. Resulting RSMD scores for each run are indicated in the lower right corner of (F–J). The run in **(G)** showed an incorrect symmetrical structure whereas the run in **(H)** showed mirroring in a vertical plane. The parameters for this experiment can be found in Supplementary Table S5.5.

When a fluorescence microscopy sample is labelled, not every potential site is labelled, and not all fluorophores will fluoresce. The degree of labelling and the performance of fluorophores strongly impacts image quality. To simulate this effect a random selection of vertex positions are not labelled with fluorophores. Results suggest that a recognizable reproduction with a good RMSD score can be obtained with up to ∼30% of the points removed (Figure 8).

### 3.3.3 Multiple Binding and Scatter

Our final noise experiment randomly chooses up to a maximum number of bound fluorophores per ground-truth point, each with a random scatter. A single ground-truth point may “spawn” up-to a maximum of individual fluorophores (*max-spawn*) using a user-set probability (*spawn-rate*). In these experiments we chose a number of parameters for “max-spawn,” “spawn-rate” and scatter.

**FIGURE 9:**
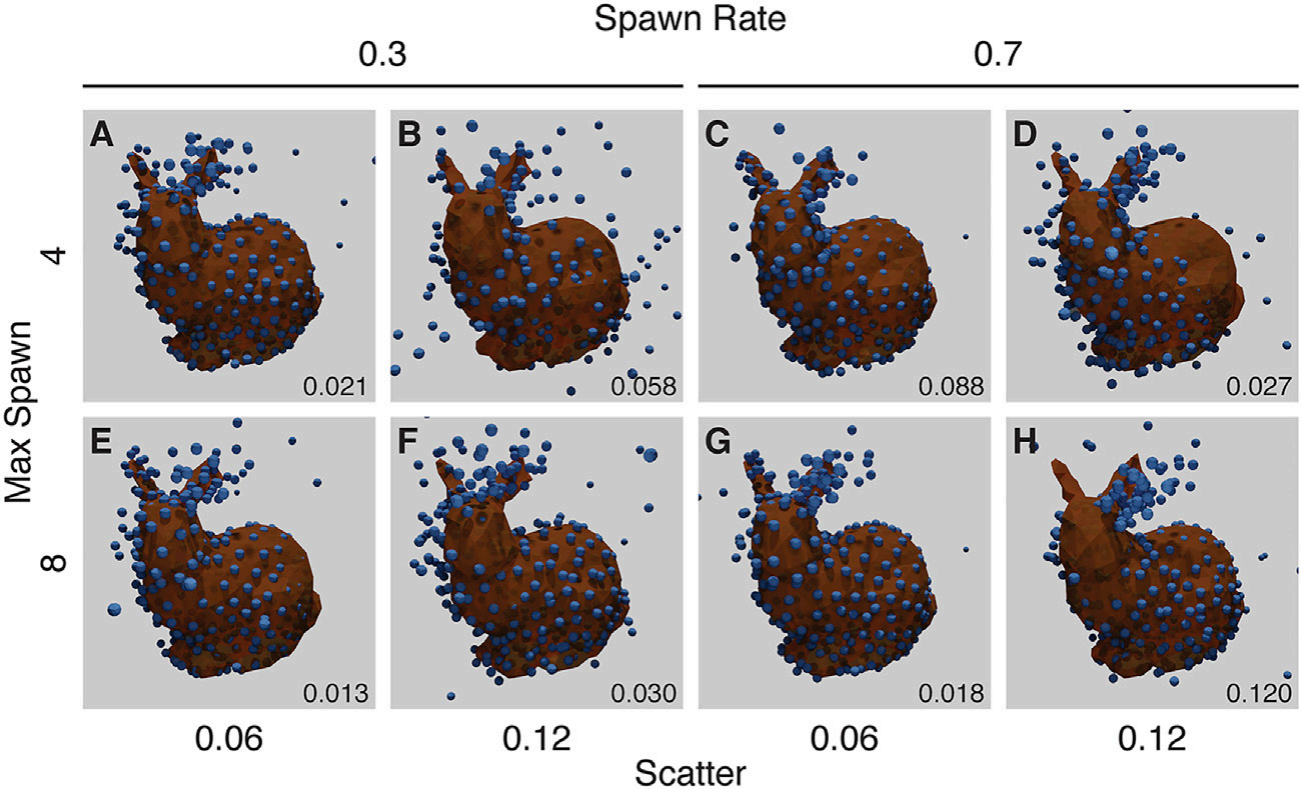
Results from the Noise Experiment for each run **(A–H)**, with resulting RMSD shown in the lower right corner of each panel. The ground truth model is shown in orange, with the created structure shown as blue spheres, overlaid and aligned. The top row **(A–D)** shows runs with a maximum number of flurophores per ground-truth point of 4, while the bottom row **(E–H)** shows runs with a maximum of 8. The left two columns **(A, B, E, F)** have a spawn-rate of 0.3, with the right two columns (C, D, G, H) have a spawn-rate of 0.7. Runs in **(A, C, D, E, F, G)** have incorrect symmetry whereas the run in **(H)** has mirroring in a vertical plane. The parameters for this experiment can be found in Supplementary Table S5.6.

Many of these runs show symmetrical structure where none should occur (Figure 9), in a manner similar to the missing fluorophores experiment (Figure 8).

These results provide additional confidence that HOLLy can produce accurate structures from experimental data. The majority of results have low RMSD scores, with identifiable structures and some tolerance to noise.

### 3.4 SMLM Dataset of the CEP152 Complex

Having optimized our approach with different 3D models, we next applied it to experimental SMLM data collected on the CEP152 complex, which is part of the centriole. One important factor with this data is that the integrated intensity varies considerably across the CEP152 data-set, with the number of localisations ranging from 5,000 to 30,000. Normalisation plays a key part in making sure this intensity range can be modelled by our network. Additionally, since this data-set is limited by the number of feasible experiments, data-augmentation plays a key role in increasing both the absolute number of training images and the variety of orientations. This training data-set consists of approximately 40,000 images, augmented from an experimental data-set of approximately 2000 images. See Supplementary Figure S2 for representative images that illustrate the range of orientations and experimental noise in this training data-set.

**FIGURE 10:**
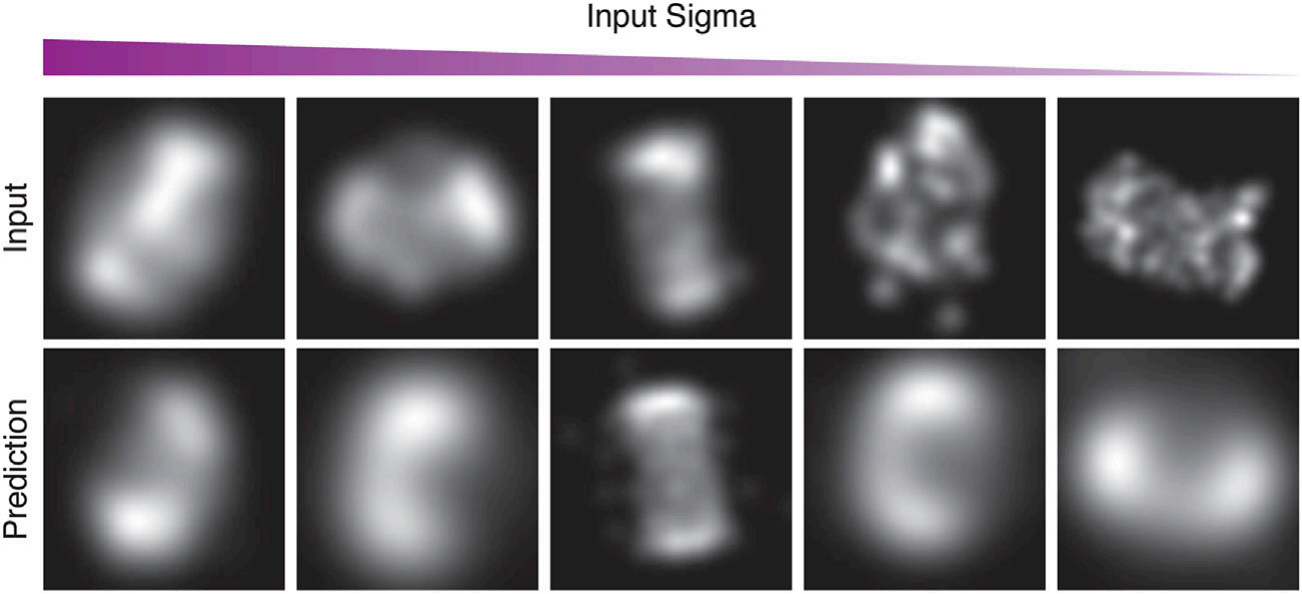
Examples from the first run of the STORM CEP152 data-set, rendered at different points during training as input-sigma values decreased. The top row shows input images from the test set. The bottom row shows the corresponding prediction.

**FIGURE 11:**
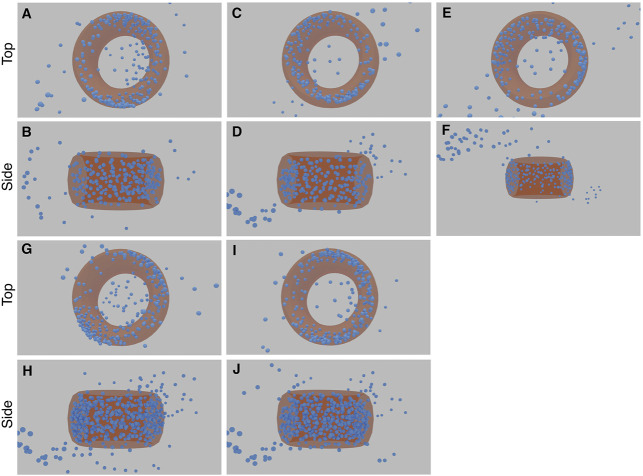
Results of the five STORM CEP152 experiments (each with a top and side view image pair). The first experiment comprises image **(A)** and **(B)**, the second experiment **(C)** and **(D)**, and so forth. The torus structure within the blue point cloud is highlighted with an orange ring. See Supplementary Material Video S1, 2 for greater clarity.

After training with these images, our network converged on a central torus for the CEP152 complex (Figures 10, 11). This inferred structure is consistent with the confirmed structure of this protein complex (Sieben et al., 2018; Kim et al., 2019).

Figure 10 in particular, shows examples of the network attempting to match the training images, both in terms of structure and the input-sigma. The input images are not completely static; recall they are generated with a particular input-sigma, which decreases as training progresses. However, the output-sigma predicted by the network does not continuously decrease as the input-sigma does—rather the rate begins to flatten towards the end of training. Indeed, certain images are rendered with a higher blur than others, suggesting that certain images are being compensated for with a higher output-sigma.

The final 3D structures in Figure 11 can be seen more easily in the videos which accompany this paper (see Supplementary Video S1, 2). When rendering these predicted structures in 2D based on the inferred orientations, they show significant blurring due to a large predicted output-sigma, even when the input-sigma was low (Figure 10). There was some noise in the inferred structure, with two “fringe-like” structures in some of the runs (Figure 11). Some points still appear in the middle of the toroidal structure, likely because the network has been unable to optimise these points as any direction they might now move in would result in an increasing error. The density appears to be lower for a small arc on the torus, reflective of the input images that also show a similar effect. These final structures are not exact as some noise still remains. Nonetheless, the consensus result that emerges from multiple runs is a toroidal structure that matches that of the CEP152 complex.

### 3.5 SIM/Expansion Microscopy Dataset of Glutamylated Tubulin in Centrioles

**FIGURE 12:**
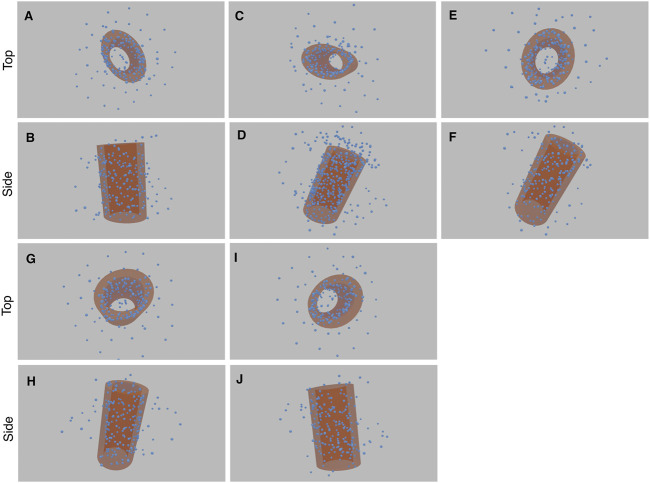
Results of the five experiments on SIM/expansion Microscopy of glutamylated tubulin in purified centrioles (each with a top and side view image pair). The first experiment comprises image **(A)** and **(B)**, the second experiment **(C)** and **(D)**, and so forth. The cylinder structure within the blue point cloud is highlighted with an orange cylinder. See Supplementary Video S3, 4 for greater clarity.

To validate our method, we also applied it to a separate experimental data-set (Mahecic et al., 2020) obtained using a different imaging technique. We analysed SIM/expansion microscopy images of glutamylated tubulin in purified centrioles. After training on the SIM/expansion microscopy images, our network converged on a central cylinder for this complex (Figure 12). The density of points is highest in the centre of each image, with a tube-like structure visible. These 3D aspects are clearer in the Supplementary Video S3, 4. There appears to be a “frill-like” structure around the top to middle of the cylinder, which may reflect a particular characteristic of the input data. Many of the images show a spike like protrusion, emanating from the top of the central cylinder (see Supplementary Figure S3).

The consensus elongated cylindrical structure produced by our method is also consistent with the known structure of glutamylated tubulin in centrioles (Mahecic et al., 2020) (See Supplementary Figure S2).

### 3.6 Handedness

Often when reconstructing 3D shapes from macroscopic images, perspective projection and occlusion effects can be used to infer depth. Neither of these are present in 2D fluorescence microscopy images. Without perspective projection, there is an unknown reflection of the final 3D geometry which cannot be determined from the data. This is known as the affine ambiguity (Hartley, 2004). Examples of this effect can bee seen in Figure 4.

## 4 Discussion

We have demonstrated a method that enables 3D structures to be reconstructed from sets of 2D SMLM or fluorescence microscopy images without any template or symmetry constraints. Our method, HOLLy, can tolerate both scatter and the limited labelling efficiency of experimental fluorescence images. The training process results in a 3D model of the structure encoded as a point-cloud in the 3D reconstruction matrix. Based on estimates of RMSD values against ground-truth and visual inspection of the results, we find that our approach can create accurate reconstructions of 3D macro-molecular structures.

Our results also demonstrate the limitations of the technique. Because of the use of 2D images, the technique is unable to resolve the chirality of the model. In addition, when the data quality is poor small structures are not reproduced. As a result when the structure is close to symmetric, the final model may become actually symmetric. On experimental data, the presence of these issues could potentially be identified by training on the same data-set multiple times and examining the differences between the results.

The value of reconstructing multiple images of a structure into a single hypothesised structure has been demonstrated in cryo-EM. In SMLM such approaches exist Heydarian et al. (2019), and show an improvement in the signal to noise ratio when combining multiple images, but performing such fits on complex structures with no constraints is extremely challenging. Here we show that, by building a 3D model and using a neural network for predicting rotation, HOLLy can discern structure from localisations with a data-set of 2000 unique images. With the increased popularity of high throughput SMLM techniques (Holden et al., 2014; Barentine et al., 2019), HOLLy provides a way to extract structural information from large volumes of super-resolution microscopy data without assumptions.

## Data Availability

The program HOLLy can be found at https://github.com/OniDaito/Holly.git. The simulated data results presented in the paper can be reproduced with the configuration files found in that repository. The STORM CEP152 complex data used in our experiments can be found on Zenodo at https://doi.org/10.5281/zenodo.4751056. Once downloaded and extracted, our results can be reproduced using the configuration file found in the HOLLy project. The GT335 tublin, centriole data can be found at https://doi.org/10.5281/zenodo.3613906. The results from out CEP152 experiments we have presented can be downloaded from Zenodo at https://zenodo.org/record/4836173.

## Footnotes

1
http://graphics.stanford.edu/data/3Dscanrep/\#bunny

2
https://www.computerhistory.org/collections/catalog/102710359

3
https://demonstrations.wolfram.com/SamplingAUniformlyRandomRotation/

4
https://pytorch.org/docs/stable/generated/torch.nn.L1Loss.html

5
http://www.cloudcompare.org/
