# Supplementary material for: 3D Structure From 2D Microscopy Images Using Deep Learning
Source: Front Bioinform. 2021 Oct 28;1:740342. doi: 10.3389/fbinf.2021.740342 (PMC9581024; doi:10.3389/fbinf.2021.740342)
Supplement: Supplementary file 2 [file DataSheet1.PDF]

Supplementary Material

1 HOLLY TECHNICAL DETAILS

Our method relies upon three major components: a convolutional neural network, a vertex tensor representing the structure used internally and a differentiable renderer. The neural network is responsible for generating the parameters required by the renderer from a batch of input images. These parameters are combined with the set of points held in the vertex tensor and passed to the renderer, whereupon an output image is produced. The differentiable renderer is a simplified 3D rasterisation pipeline of transformation matrices, though the perspective matrix is omitted as our microscopy images do not show any apparent change due to distance from the camera. Several advanced, differentiable renderers already exist though they are designed to render polygons instead of Gaussian-blurred points. This set of transformation matrices is completely differentiable allowing for error gradients to pass backward from the loss function through the pipeline.

Neural networks learn through back-propagating the error (or loss) between the expected output and the generated output. This loss is produced by a loss function - typically the Least Squared Error (or L2) loss. We chose the Least Absolute Deviations (or L1) loss on the pixel values of the input and output image batches. L1 was chosen as it has been shown to outperform the more common L2 on pixel comparison tasks. The loss is sent to the Adaptive Moment Estimation (Adam) stochastic optimiser (Kingma and Ba, 2017) in order to update the network weights and the positions of the points in our structure set.

Our network consists of 10 convolutional layers and two fully connected layers. Leaky ReLU (Rectified Linear Unit) is the activation function used at each layer except the final output layer. The output layer comprises the rotational and translational parameters, and in certain experiments, the predicted sigma value, discussed in the experiment section. Each convolutional layer uses batch normalisation (Ioffe and Szegedy, 2015). The first convolutional kernel is 5 by 5 pixels, with all subsequent kernels being 3 by 3 pixels. A padding size of 2 and a stride of 2 are used. After the first layer, the subsequent layers come in pairs - the first having a stride of two, the second having a stride of 1. This performs a similar operation to a pooling layer. The final convolutional layer performs a final pooling with it's stride of 2.

The vertex tensor consists of a number of points in 3D space, represented in homogeneous coordinates (x, y, z and w). The size of this set is chosen by the user to best match the integrated density of the input data. When no normalisation strategy is applied, each individual point has an intensity of 1. Network performance is highly dependent on the size of this set. Therefore there is a trade-off between model accuracy and speed, with memory requirements scaling significantly with the number of points requested. The number of points is a so-called 'hyper-parameter', along with other hyper-parameters such as the learning rate and batch size.

The network predicts between 3 and 6 continuous parameters - depending on the options chosen by the user. The orientation is represented in 'Angle-Axis' format: 3 real numbers for the rotation axis with the magnitude representing the angle. These are unchanged and passed to the renderer directly from the neural network. Translation in the X-Y plane is restricted via a *tanh* function, which has the effect of limiting very large movements. Translation is restricted to -0.1 to 0.1. The final parameter is the sigma value used to render the output images. Output sigma is clamped to a maximum of 14 and is passed through a Softplus function.

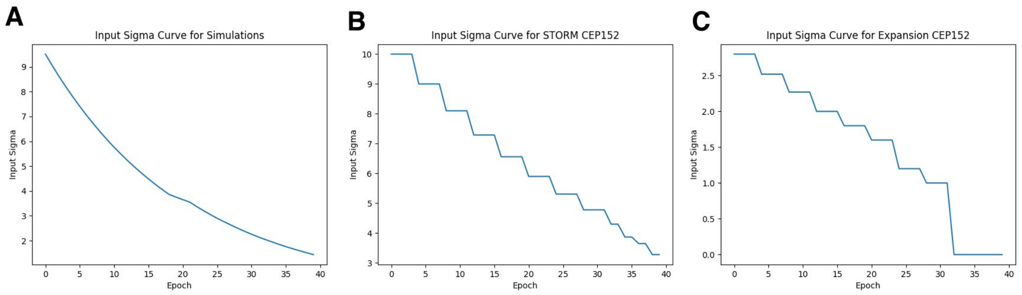

Figure S1. The input-sigma curve used in our experiments with simulated data models (A), STORM CEP152 data (B) and Expansion Microscopy CEP152 (C). The input-sigma in image C is the level of blur applied to images that already contain some unknown level of blur. The final value of 0 in image C is intentional; the images are being use with no additional blur.

The output of the renderer is a batch of images. Each fluorophore in the image is represented as a 2D Gaussian with a particular sigma (Equation S1). The input data is rendered with a sigma value chosen by the user. The output data generated by the network can either have its sigma value derived by the network or also set by the user. A large sigma - for example 10.0 - results in a very blurry image, with each point having a Full Width at Half Maximum (FWHM) of 138 pixels, whereas a sigma of 1.2 has a FWHM of 2 pixels. The network begins training on images rendered with a large sigma, progressing to a smaller sigma as training continues. This leads to better convergence as large changes in pixel intensity across the image are not present, providing smoother gradients over large distances. Initially, points are able to move further, refining their positions with smaller movements later in the training.

$$G(x,y) = \frac{1}{2\pi\sigma^2} e^{-\frac{x^2+y^2}{2\sigma^2}} \tag{S1}$$

2 CONVERTING MATLAB SMLM DATA

The program used to convert the experimental STORM CEP152 data into a series of FITS images can be found on GitHub[1]. It uses the same rendering pipeline as the differentiable renderer found in HOLLy.

Generating a set of training images to replicate our results takes roughly 3 days on a AMD Ryzen Threadripper 1920X (24) running at 3.500GHz.

3 INPUT-SIGMA HYPER-PARAMETER.

The two input-sigma profiles are shown in Figure S1. We generated images on demand through training when using simulated point-clouds, therefore the input curve can be smoothly altered per training step as opposed to per-epoch. However, the experimental CEP152 data was rendered with discrete sigma values, therefore has a 'stepped' curve.

4 FINAL STRUCTURE VIDEOS.

To aid in visualising the final 3D structure of the STORM CEP152 complex as discerned by HOLLy, we have included a number of videos that show the final structure for each of the 5 experiments (Video S1), including the progression through training (Video S2).

[1] `https://github.com/onidaito/ceprender/`

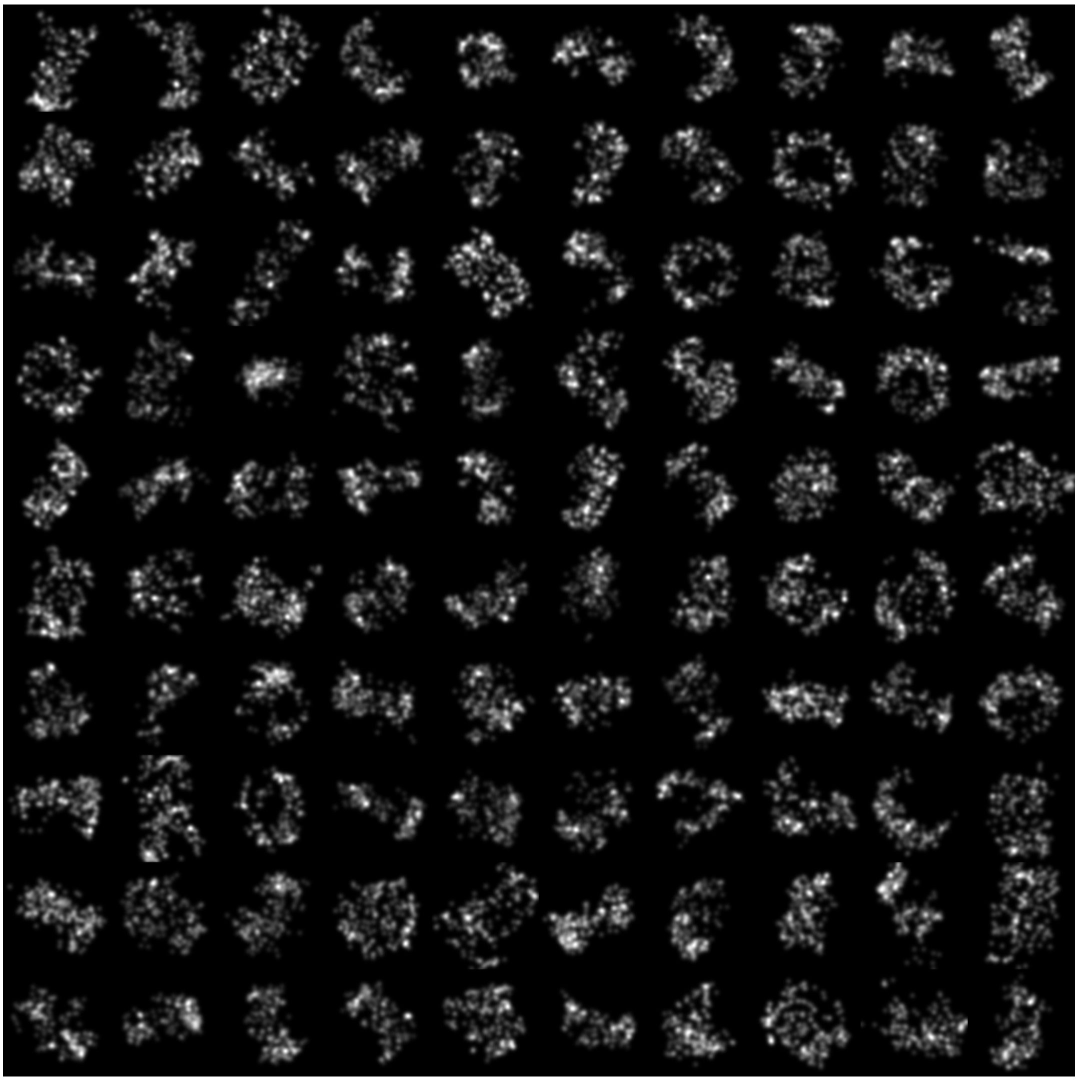

Figure S2. A random selection of 100 images from the STORM CEP152 dataset, rendered with an input sigma of 2.52. This small sample is broadly representative of the 40,000 individual images within the training dataset.

Similarly, to aid in visualising the final 3D structure of the Expansion Microscopy Centrioles as discerned by HOLLy, we have included a number of videos that show the final structure for each of the 5 experiments (Video S3), including the progression through training (Video S4).

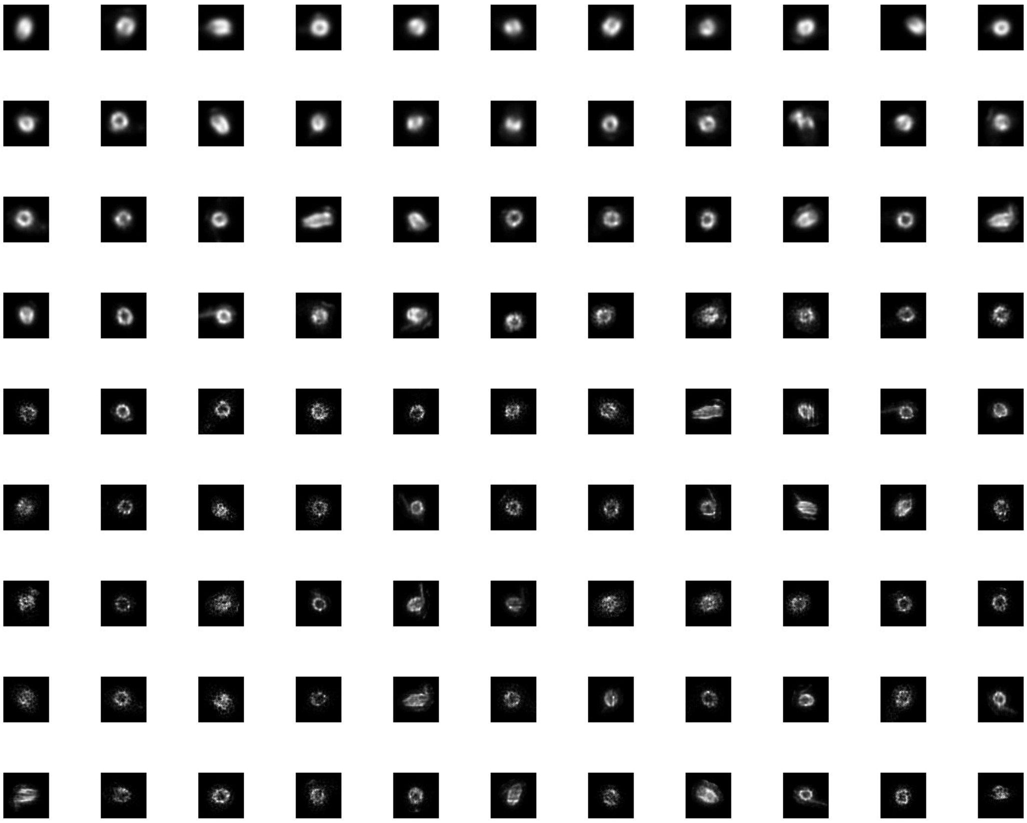

Figure S3. A random selection of 100 images from the Expansion Microscopy Centriole dataset. This small sample is broadly representative of the 14,000 individual images within the training dataset. Note that these images are much smaller than these used in the simulated or STORM experiments.

5 EXPERIMENT PARAMETERS

The key parameters for every experiment are listed below. Example parameters for the baseline experiments are included in the code-base, available online (see Code Availability Statement).

5.1 Stanford Bunny Experiment parameters

Run	Epochs	Learning Rate	Num. of Points	Max. Input Sigma	Min Input Sigma
1.A to 1.E	40	0.0004	350	10	1.41

5.2 Utah Teapot Experiment parameters

Run	Epochs	Learning Rate	Num. of Points	Max. Input Sigma	Min Input Sigma
2.A to 2.E	40	0.0004	230	10	1.41

5.3 CEP152 Approximation Experiment parameters

Run	Epochs	Learning Rate	Num. of Points	Max. Input Sigma	Min Input Sigma
3.A to 3.E	40	0.0004	180	10	1.41

5.4 Scatter Experiment parameters

Run	Epochs	Learning Rate	Num. of Points	Scatter	Max. Input Sigma	Min Input Sigma
4.A	40	0.0004	350	0.03	10	1.41
4.B	40	0.0004	350	0.06	10	1.41
4.C	40	0.0004	350	0.09	10	1.41
4.D	40	0.0004	350	0.12	10	1.41
4.E	40	0.0004	350	0.15	10	1.41

5.5 Drop-out Experiment parameters

Run	Epochs	Learning Rate	Num. of Points	Drop-out	Max. Input Sigma	Min Input Sigma
5.A	40	0.0004	350	0.1	10	1.41
5.B	40	0.0004	350	0.3	10	1.41
5.C	40	0.0004	350	0.5	10	1.41
5.D	40	0.0004	350	0.7	10	1.41
5.E	40	0.0004	350	0.9	10	1.41

5.6 Spawn Experiment parameters

Run	Epochs	Learning Rate	Num. of Points	Drop-out	Scatter	Max Spawn	Spawn Rate
6.A	40	0.0004	350	0.1	0.06	4	0.3
6.B	40	0.0004	350	0.1	0.12	4	0.3
6.C	40	0.0004	350	0.1	0.06	4	0.6
6.D	40	0.0004	350	0.1	0.12	4	0.6
6.E	40	0.0004	350	0.3	0.06	4	0.3
6.F	40	0.0004	350	0.3	0.12	4	0.3
6.G	40	0.0004	350	0.3	0.06	4	0.6
6.H	40	0.0004	350	0.3	0.12	4	0.6

6 DIRECT OPTIMISATION

We tested whether or not we could directly optimise the pose parameters with no convolutional layers. We used the Utah Teapot and Stanford bunny models, no sigma prediction and the following parameters:

Run	Model	Epoch	Learning Rate	Num. of Points	Max. Input S.	Min Input S.	Optimiser
7.A	teapot	100	0.0004	250	10	1.41	Adam
7.B	teapot	100	0.004	250	10	1.41	Adam
7.C	bunny	100	0.0004	350	10	1.41	Adam
7.D	bunny	100	0.004	350	10	1.41	Adam
7.E	teapot	100	0.0004	250	10	1.41	SGD
7.F	teapot	100	0.004	250	10	1.41	SGD
7.G	bunny	100	0.0004	350	10	1.41	SGD
7.H	bunny	100	0.004	350	10	1.41	SGD

We also performed a run with noise added, using the following parameters:

Run	Spawn Rate	Scatter	Epoch	L. Rate	Num. of Points	Max. Input S.	Min Input S.	Optimiser
7.I	0.3	0.06	100	0.0004	350	10	1.41	SGD

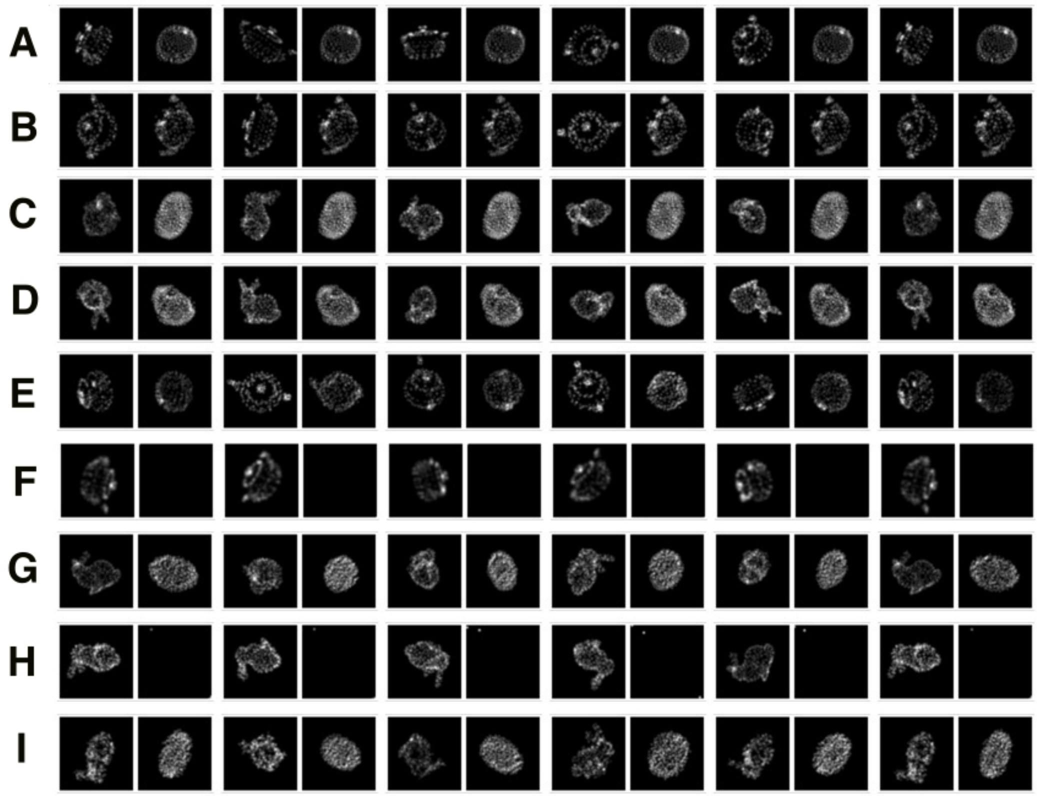

Figure S4. The final results from runs 7.A to 7.I - direct optimisations. Each row contains 6 pairs of images. The first image in each pair is the input image - the second is the corresponding output. Run 7.B appears to show a derived structure similar to the ground-truth, though this is misleading as the model only appears reasonable from one angle (viewing the final model in 3D shows this more clearly). Runs 7.F and 7.H fail to create any structure. Runs 7.E, 7.G and 7.I show some response to the different input rotations, whereas the remaining runs show very similar output poses.

REFERENCES

Ioffe, S. and Szegedy, C. (2015). Batch Normalization: Accelerating Deep Network Training by Reducing Internal Covariate Shift. In *International Conference on Machine Learning* (PMLR), 448–456

Kingma, D. P. and Ba, J. (2017). Adam: A Method for Stochastic Optimization. In *3rd International Conference on Learning Representations,{ICLR} 2015, San Diego, CA, USA*
